# Bi-Nitrobenzene Poisoning

**Published:** 1910-08-20

**Authors:** 


### Toxicology.

### BI-NITROBENZENE POISONING.

The number of more or less toxic organic compounds that are to be met with commercially is constantly increasing, and it is scarcely possible for text-books of toxicology to keep pace with them. It may easily happen that a patient may be suffering from the effects of some compound used by him at his work, and yet for the fact to pass unrecognised. The case recorded by Dr. Edward Walker in the *British Medical Journal* is an instance in point; the symptoms were extremely like those of acute yellow atrophy of the liver, but the conclusion come to was that the patient was killed by bi-nitrobenzene poisoning. In any case, it is right that the symptoms should be well known by those who practise in towns where bi-nitrobenzene is used, lest other cases of the same sort should occur. Moreover, it seems well worth while investigating the actions of bi-nitrobenzene experimentally with a view to throwing more light upon the obscure disease known as acute yellow atrophy of the liver. From the general features of Dr. Walker's case it seems quite possible that acute yellow atrophy of the liver was produced by the toxic action of bi-nitrobenzene.

The patient was a lad of 16, who first came under Dr. Walker's observation at Huddersfield Infirmary on May 25, 1907. The full history was not obtained at once; but it was ultimately found that he had been working for some time at a chemical works where he came into contact with bi-nitrobenzene between January and April 1907. During that time he had twice been ill and away from work for a couple of days suffering from toxic symptoms that were attributed to the effects of bi-nitrobenzene. On April 29 a third attack of the same kind began, but it proved much more serious and ended in death. He was taken ill with a sense of weakness, dizziness, and with unsteadiness in his legs, associated with shortness of breath, pallor of the cheeks, and some lividity of the lips and fingers. Having stayed away from the works for a few days he became sufficiently better to return to a different job—in the open air. He was not in his usual good health, however, and was always fagged out by the evening; besides which he began to suffer cramps in the legs, and to vomit dark-green material upon one or two occasions. On May 19 he went home very much worse than usual; he may be said to have entered upon the final phase of his illness on this day. He was very sick, dizzy, and light-headed, with pains in his head and limbs, and his urine was like porter. His skin and conjunctivæ began to be jaundiced, and progress was steadily downhill. On May 25, on admission to the infirmary, he was pale and bluish, though the blueness did not amount to cyanosis. He was very weak and prostrate. His skin was a dirty yellow, and his conjunctivæ were also yellow. He was restless, short of breath, rambling, excited, and sleepless. There was no pyrexia. The pulse was 80, feeble, and of low tension. The tongue was dry and coated, the palate yellowish, and the gums blue. There was marked tenderness over the liver, stomach, and spleen, with constipation and bilious vomiting. The urine was rich dark-brown in colour, of specific gravity 1,020; it was clear and of faintly acid reaction. No sugar was present, and there was very little bile pigment, but a great deal of urobilin both to the chemical and to the spectroscopic tests and quantities of indican. The yellow pigmentation on the hands, constantly found in workers amongst bi-nitro compounds, and persisting in this case for more than four weeks after the last contact with them, would help to suggest the diagnosis if the history of work amongst these substances was not spontaneously forthcoming. When seen, Dr. Walker's patient was in no condition to give any account of the history of his illness.

After admission he showed no sign of improvement. Each day the "jaundice" deepened. Rambling and excitement passed on to coma, and death resulted upon May 29.

If the diagnosis had been acute yellow atrophy of the liver, the lesions found at the autopsy would have been in many respects confirmatory. The liver weighed only 28 ounces, and it was generally paler than usual, especially the right lobe. In consistence it was soft, and so friable that it was readily broken down. On section the whole of its interior had a peculiar yellowish-green mottling. The gall-bladder was not empty, however, but contained about 6 ounces of dark thick bile. The points upon which Dr. Walker lays stress in terming the case one of bi-nitrobenzene poisoning, and not one of acute yellow atrophy of the liver, are the facts that the gall-bladder was not empty, that none of the internal viscera exhibited bile staining, that neither leucin nor tyrosin were to be found in the urine, and that the history of former attacks whilst in the works pointed directly to bi-nitrobenzene being the cause of the trouble. It might be added that the state of the kidneys is suggestive of the action of a toxin also—each weighed 7 ounces, and was pale and mottled.

